# An Expectation-Maximization Algorithm for Including Oncological COVID-19 Deaths in Survival Analysis

**DOI:** 10.3390/curroncol30020163

**Published:** 2023-02-08

**Authors:** Francesca De Felice, Luca Mazzoni, Franco Moriconi

**Affiliations:** 1 Department of Radiological Science, Oncology and Human Pathology, “Sapienza” University of Rome, Policlinico Umberto I, 00161 Rome, Italy; 2 Alef—Advanced Laboratory Economics and Finance, 00198 Rome, Italy; 3 Department of Economics, University of Perugia, 06123 Perugia, Italy

**Keywords:** COVID-19, survival analysis, kaplan-meier estimator, informative censoring, extended greenwood’s formula, em algorithm, mean-imputation

## Abstract

We address the problem of how COVID-19 deaths observed in an oncology clinical trial can be consistently taken into account in typical survival estimates. We refer to oncological patients since there is empirical evidence of strong correlation between COVID-19 and cancer deaths, which implies that COVID-19 deaths cannot be treated simply as non-informative censoring, a property usually required by the classical survival estimators. We consider the problem in the framework of the widely used Kaplan–Meier (KM) estimator. Through a counterfactual approach, an algorithmic method is developed allowing to include COVID-19 deaths in the observed data by mean-imputation. The procedure can be seen in the class of the *Expectation-Maximization* (EM) algorithms and will be referred to as *Covid-Death Mean-Imputation* (CoDMI) algorithm. We discuss the CoDMI underlying assumptions and the convergence issue. The algorithm provides a completed lifetime data set, where each Covid-death time is replaced by a point estimate of the corresponding virtual lifetime. This complete data set is naturally equipped with the corresponding KM survival function estimate and all available statistical tools can be applied to these data. However, mean-imputation requires an increased variance of the estimates. We then propose a natural extension of the classical Greenwood’s formula, thus obtaining expanded confidence intervals for the survival function estimate. To illustrate how the algorithm works, CoDMI is applied to real medical data extended by the addition of artificial Covid-death observations. The results are compared with the estimates provided by the two naïve approaches which count COVID-19 deaths as censoring or as deaths by the disease under study. In order to evaluate the predictive performances of CoDMI an extensive simulation study is carried out. The results indicate that in the simulated scenarios CoDMI is roughly unbiased and outperforms the estimates obtained by the naïve approaches. A user-friendly version of CoDMI programmed in R is freely available.

## 1. Introduction

The problem of defining a common and appropriate method in survival analysis for handling the dropouts due to coronavirus disease 2019 (COVID-19) deaths of patients participating to oncology clinical trials has been recently stressed [1,2]. In oncology trials, all-causality deaths are often counted as events for death-related endpoints, e.g., overall survival. However, as it has been pointed out [2], counting a COVID-19 fatality as a death-related endpoint requires a complex redefinition of the estimand, considering a composite strategy for using the so-called *intercurrent events* [3], as, e.g., “discontinuation from treatment due to COVID-19” or “delay of scheduled intervention”. The problem is also exacerbated by the difficulty of homogeneously determining whether a death is entirely attributable to COVID-19. In this paper, we address a simplified version of this problem, assuming that COVID-19-related deaths are homogeneously identified and are the only intercurrent events to be considered. In this framework, we tackle the problem of how data in an oncology trial having the overall survival as the endpoint can be dealt with when deaths due to COVID-19 are present in the sample.

COVID-19 deaths should not be treated as standard censored data, because usual censoring should be considered—at least in principle—*non-informative*. Informative censoring, instead, occurs when participants are lost to follow-up also due to reasons related to the study, as it seems to be the case with COVID-19 deaths of oncological patients. Direct data on how COVID-19 affects survival outcomes in patients with active or a history of malignancy are immature. However, early evidence identified increased risks of COVID-19 mortality in patients with cancer, especially in those patients who have progressive disease [4]. Patients with cancer and COVID-19 were associated with increased death rate compared to unselected COVID-19 patient population (13% versus 1.4%) [4,5]. Based on these findings, in survival analysis dropouts due to COVID-19 deaths should be considered as cases of *informative censoring*. Another way used in survival analysis literature to represent this dependence is to view cancer deaths and COVID-19 deaths as *competing events*, see, e.g., [6] Ch. 8. In this paper, we propose an algorithmic method to include COVID-19 deaths of oncological patients in typical survival data, focusing on the classical Kaplan–Meier (KM) product-limit survival estimator. Our method is in the spirit of the *Expectation-Maximization* (EM) algorithms [7] used for handling missing or fake data in statistical analysis. In this sense, the method could also be used in applications different from clinical trials, e.g., reliability analysis. Correction of actuarial life tables can be also a possible application.

An overview of methods for dealing with missing data in clinical trials is provided by DeSouza, Legedza and Sankoh [8]. See also Shih [9]. In Shen and Chen [10] the problem of doubly censored data is considered and a maximum likelihood estimator is obtained via EM algorithms that treat the survival times of left censored observations as missing. As concerning situations with informative censoring, where there is stochastic dependence between the time to event and the time to censoring (which is our case if “censoring” is a COVID-19 death), a distinction is proposed by Willems, Schat and van Noorden [11] among cases where the stochastic dependence is *direct*, or *through covariates*. In that paper [11], the latter case is considered and an “inverse probability censoring weighting” approach is proposed for handling this kind of censoring. Since at this stage it is difficult to model cancer deaths and COVID-19 deaths through covariates in common, in this paper, we consider the case of direct dependence. We do not consider a survival regression model based on specified covariates, and limit the analysis, as has been said, to the basic Kaplan–Meier survival model, which is assumed to be applied, as usual, to a sufficiently homogeneous cohort of oncological patients. In this framework, we propose a so-called *mean-imputation* method for COVID-19 deaths using a purpose-built algorithm, referred to as *Covid-Death Mean-Imputation* (CoDMI) algorithm. A user-friendly version of this algorithm programmed in R is freely available. The corresponding source code can be downloaded from the website: https://github.com/alef-innovation/codmi (accessed on 6 July 2021).

An alternative approach to survival analysis when COVID-19 deaths are present in an oncology clinical trial in addition to cancer deaths could be based on the *cumulative incidence functions*, which estimate the marginal probability for each competing risks.This would lead to dealing with subdistributions and would require appropriate statistical tests to be used, see, e.g., [12]. Our algorithmic approach, instead, acts directly on the data, producing an adjustment that virtually eliminates the presence of the competing risk, thus allowing the use of standard statistical tools. This comes at the price of accepting some simplifications and specific assumptions.

The basic idea of CoDMI algorithm is of a counterfactual nature. Since the KM model provides an estimation of the probability distribution to survive until a chosen point on the time axis for any patients in the sample, for each of the patients which is observed to die of COVID-19 at time θ, we derive from this distribution ${\hat{e}}_{\theta }$, the expected lifetime beyond time θ, thus obtaining the “no-Covid” expected lifetime $\hat{\tau }=\theta +{\hat{e}}_{\theta }$ for each of these patients. Each θ value is then replaced by the virtual lifetime $\hat{\tau }$ (this is the mean-imputation) and the KM estimation is repeated on the original data completed in this way, providing a new estimate of $\hat{\tau }$. This procedure is iterated until the change between two successive $\hat{\tau }$ estimates is considered immaterial (according to a specified criterion).

It is pointed out by Shih [9] that “The attraction of imputation is that once the missing data are filled-in (imputed), all the statistical tools available for the complete data may be applied”. Although in our case we are not dealing with missing data but with partially observed data, this attractive property of mean-imputation still holds true. It should be noticed, however, that in general, treating an *estimated* value—even an unbiased one—as an *observed* value should require some increase in variance. In particular, the confidence limits of KM estimates on data including imputations should be appropriately enlarged. We propose an extension of the classical Greenwood’s formula providing this correction.

The paper is organized as follows. In Section 2, the notations and the basic structure of the KM survival estimator are provided and the related problem of computing expected lifetimes is illustrated. The representation of Covid-death cases in the sample is described. In Section 3, CoDMI algorithm is introduced and the details of the iteration procedure are provided. The convergence issue is discussed and the underlying assumptions of the algorithm are considered, taking into account some subtleties required by the non-parametric nature of the KM estimator. A possible *adjustment for censoring* of the algorithm is presented and a correction of Greenwood’s formula is derived for taking into account the estimation error in the imputed point estimates. Application of CoDMI to real medical data are provided in Section 4. Two oncological survival data sets which are well referenced in the literature are completed by artificial Covid-death observations and the survival curves estimated by CoDMI are compared with the no-Covid KM estimates and with the two naïve KM estimates obtained by considering COVID-19 deaths as censorings or as death of disease. The effect of the final adjustment for censoring is also illustrated. In Section 5, an extensive simulation study is presented to evaluate the CoDMI predictive performances. We discuss the details of the simulation procedure and provide tables illustrating the results. Some conclusions and comments are given in Section 6. In Appendix A a derivation of the extended Greenwood’s formula is provided.

## 2. Notation and Assumptions on Covid Deaths in the Sample

### 2.1. Typical Clinical Trial Data and the Kaplan–Meier Estimator

We consider a study group of *n* oncological patients which received a specified treatment and are followed-up for a fixed calendar time interval. The response for each patient is the survival time $T_{0}$ which is computed starting from the date of enrollment in the study, date 0.

**Remark** **1.** *This is in line with the standard actuarial notation, where $T_{x}$ is used to denote the survival time of a subject of age x. Our patients actually have “age 0” (in the study) at the time they are enrolled.*

Typically, the observations include *censored* data, that is, survival times known only to exceed reported value. Formally, for a given patient there is a censoring at time point *t* if we only know that for this patient $T_{0}>t$. If $t_{\max}$ denotes the last observed time point in the study, i.e., $t_{\max}$ corresponds to the current date or the end of the study, the case of a censored time $t<t_{\max}$ corresponds to a patient *lost to follow-up*. To take into account censoring, the observations can be represented in the form:

$$\left\{z_{i}=\left(t_{i},d_{i}\right),\;i=1,\cdots ,n\right\}\;,$$

where $t_{i}$ is the observed survival time of patient *i* and $d_{i}$ is a “status” indicator at $t_{i}$ which is equal to 1 if *death of disease under study* (DoD) is observed and is equal to 0 if there is a censoring (Cen) on that time. We assume that the group of patients provides a homogeneous sample, that is, all the observations $t_{i}$ come from the same probability distribution for $T_{0}$, and our aim is to estimate the cumulated probability function $F\left(t\right)=P\left(T_{0}<t\right)$, or the related survival function:

$$S\left(t\right)=1-F\left(t\right)=P\left(T_{0}\geq t\right)\;.$$

The estimation of S(t) can be realized non-parametrically by the well-known Kaplan–Meier product-limit estimator [13]. If we denote by:

$$z_{\left(i\right)}=\left(t_{\left(i\right)},d_{\left(i\right)}\right),\;i=0,1,\cdots ,n\;,$$

the observations $z_{i}$ ordered by increasing value of *t* (with $t_{\left(0\right)}=d_{\left(0\right)}=0$), the KM estimator is written as:

(1) $$\hat{S}\left(t\right)=\prod_{i:t_{\left(i\right)}\leq t}\left(1-\frac{d_{\left(i\right)}}{R(t_{\left(i\right)})}\right)\;,$$

where $R(t_{\left(i\right)})$ is the number of subjects at risk at (immediately before) time $t_{\left(i\right)}$, and the ratio $h\left(t_{\left(i\right)}\right)=d_{\left(i\right)}/R\left(t_{\left(i\right)}\right)$ is the *hazard rate* at time $t_{\left(i\right)}$. Therefore $\hat{S}\left(t\right)$ is a (left continuous) step function with steps at each time a DoD event occurs.

**Remark** **2.** *(i) If there are ties in the sample, the ordering can always be unambiguously defined by adopting the appropriate conventions. We refrain here from describing these conventions, already considered in the original paper* [13] *and extensively discussed in the subsequent literature.*

*(ii) In general, the event of interest (in our case DoD) acts on the ratio $d_{\left(i\right)}/R(t_{\left(i\right)})$ in the estimator* (1) *by modifying both the numerator and the denominator. The not-of-interest event (Cen) only acts on the denominator. This follows from the assumption that a Cen corresponds to a non-informative censoring.*

It is assumed that the censored observations do not contribute additional information to the estimation, which is the case if censoring is independent of the survival process. If the time points $t_{i}$ are given, it was already shown in the original paper [13] that (1) is a maximum likelihood estimator. Obviously $t_{\left(n\right)}=t_{\max}$, the last time point in the observed sequence. For our purposes, it is important to distinguish two cases, depending on whether at $t_{\max}$ there is a DoD or a Cen.

### 2.2. The Case of Complete Death-Observations

If $d_{\left(n\right)}=1$, i.e., $t_{\max}$ relates to a DoD event, and if $R(t_{\max})=1$, then one has $S(t_{\max})=0$, which means that the data allows us to estimate the entire probability distribution of $T_{0}$. Let us refer to this case as the *complete death-observations case* or, briefly, the *complete case*. In this situation, we can compute the estimated expected future lifetime for a patient which is alive at time θ≥0. Let us denote the *conditional lifetime*, given θ, as:

$$T_{\theta }=T_{0}|\left(T_{0}\geq \theta \right)\;.$$

Then the *expected future lifetime* (the *life expectancy*) beyond θ is:

(2) $${\hat{e}}_{\theta }:=\hat{E}\left(T_{\theta }\right)-\theta =\frac{1}{\hat{S}\left(\theta \right)}\;\int_{\theta }^{t_{\left(n\right)}}\hat{S}\left(t\right)dt\;.$$

Since $\hat{S}\left(t\right)$ is a step function and the jump at time $t_{\left(i\right)}$ with $d_{\left(i\right)}=1$ equals the probability $q_{\left(i\right)}$ to die of disease at this time point, (2) is equivalent to the average taken on the *truncated distribution* of $T_{0}-\theta$:

(3) $${\hat{e}}_{\theta }=\frac{\sum_{i:t_{\left(i\right)}>\theta }\left(t_{\left(i\right)}-\theta \right)\;q_{\left(i\right)}}{\sum_{i:t_{\left(i\right)}>\theta }\;q_{\left(i\right)}}\;,$$

where $q_{\left(i\right)}=0$ if $d_{\left(i\right)}=0$.

### 2.3. The Incomplete Case

If the condition $d_{\left(n\right)}=1$ is not fulfilled, we are in an *incomplete* (*death-observations*) *case*: one has $S(t_{\max})>0$, meaning that the data are not sufficient to estimate the entire survival distribution, then the expected future lifetime $e_{\theta }$ cannot be derived without some ad hoc choices or suitable additional assumptions.

Let us denote by $t_{\max}^{\left(D\right)}$ the last observed time point of a DoD event (i.e., $t_{\max}^{\left(D\right)}=\max\left\{t_{i}:\;d_{i}=1\right\}$). If $t_{\max}>t_{\max}^{\left(D\right)}$, the KM estimate only provides the final survival probability $Q_{\mathrm{fin}}=\hat{S}\left(t_{\max}^{\left(D\right)}\right)>0$. We then choose to complete the distribution by setting $\hat{S}\left(t_{\max}\right)=0$, which is equivalent to posing the entire probability mass $Q_{\mathrm{fin}}$ on the last time point $t_{\max}$. In terms of the data, this is also equivalent to change to 1 the status indicator $d_{\left(n\right)}$. The effect of this choice depends on the actual meaning we attribute to the random variable $T_{0}$. If $T_{0}$ represents the entire future lifetime of the patients since they entered the study, then posing $\hat{S}\left(t_{\max}\right)=0$ provides an underestimation of the life expectancy, since we have $\theta +e_{\theta }\leq t_{\max}$ while we know that at least one patient was alive at the end of the study. In many cases, however, it is convenient to assume that the variable of interest is the patient’s lifetime *in the study*. Formally, we would consider the random variable $T_{0}^{\prime }=\min\left\{T_{0},t_{\max}\right\}$, where $t_{\max}$ is the duration of the study. The completed survival function refers to this random variable and no underestimation would be produced in this case. This issue is strictly related to the special nature of the final time point $t_{\max}$ in this kind of survival problems. For example, *self-consistency*, an important property of the KM estimator, only holds if $\hat{S}\left(t_{\max}\right)=0$

**Remark** **3.** *This was pointed out by Efron* [14] *p. 843, where it is observed that the iterative construction underlying the KM estimator “sheds some light on the special nature of the largest observation, which the self-consistent estimator always treats as uncensored, irrespective of” $d_{\left(n\right)}$.*

### 2.4. Including Covid-Death Events in the Data

Assume that, in addition to the *n* patients who left the study by a DoD or a Cen event, also *m* patients were present in the oncological trial for whom *death of COVID-19* (DoC) was observed on the time points $\theta_{j},\;j=1,\cdots ,m$. The corresponding observed data set can be represented as follows:

(4) $$x=z\cup \theta =\left\{z_{i}=\left(t_{i},d_{i}\right),\;i=1,\cdots ,n\right\}\cup \left\{(\theta_{j},\cdot ),\;j=1,\cdots ,m\right\}\;,$$

where the status indicator of each DoC event is missing. It is clearly inappropriate to pose these indicators equal to 1, but it is also not appropriate to set them equal to 0, since the DoC event provides an informative censoring, given that we know this event does carry prognostic information about the survival experience of the oncological patients. More precisely, we know that there is a positive correlation between DoD and DoC events. However, ignoring DoC data would cause an unpleasant loss of information and we would like to adjust these data in some ways, so that it can be included in the study. Formally, we are interested in replacing each of the observed $\theta_{j}$ by a different appropriate time point $\tau_{j}>\theta_{j}$, a *virtual lifetime* conditional on $\theta_{j}$, possibly with an appropriate value of the corresponding status indicator, which we will denote by $\delta_{j}$. We are confident that this replacement of the DoC time points can be properly completed just because we assume that, due to the dependence between DoC and DoD events, the “standard” data *z* contain information on the COVID-19 data (and vice versa). The determination of the status indicators $\delta_{j}$ is more challenging. However, with the appropriate adjustment we can consider the whole data set:

(5) $$w=\left\{z_{i}=\left(t_{i},d_{i}\right),\;i=1,\cdots ,n\right\}\cup \left\{z_{j}^{\prime }=\left(\tau_{j},\delta_{j}\right),\;j=1,\cdots ,m\right\}\;,$$

and we can safely apply the KM estimator to these data, thus also using the information contribution carried by COVID-19 deaths. In the following section, we will propose an iterative procedure to suitably realize this adjustment.

## 3. The EM Mean-Imputation Procedure

### 3.1. The CoDMI Algorithm

Obviously, the input data to the algorithm are given by the observation set *x* in (4). We will assume, however, that all patients who died of COVID-19 would have died of disease if COVID-19 had not intervened, thus setting $\delta_{j}\equiv 1$, i.e., assuming that all the virtual lifetimes $\tau_{j}$ would have been terminated by a DoD event). We will see in Section 3.4 how one can try to get around this limitation in this counterfactual problem. Under the assumption $\delta_{j}\equiv 1$, the basic idea of our COVID-19 adjustment is to estimate the virtual lifetimes $\tau_{j}$ as the expectation $E(T_{\theta_{j}})$, provided by the KM estimator itself. This is realized by a procedure consisting of the following steps.

- *Initialization step*. One starts by setting $\left(\tau_{j},\delta_{j}\right)=\left({\hat{\tau }}_{j}^{\left(0\right)},1\right)$ for j=1,2,⋯,m, where ${\hat{\tau }}_{j}^{\left(0\right)}$ are arbitrarily chosen initial values. Then one obtains an artificial complete data set ${\hat{w}}^{\left(0\right)}$, as defined in (5). Examples of initialization are ${\hat{\tau }}_{j}^{\left(0\right)}\equiv \theta_{j}$ or ${\hat{\tau }}_{j}^{\left(0\right)}\equiv \theta_{j}+{\hat{e}}_{\theta_{j}}^{\left(z\right)}$, where ${\hat{e}}_{\theta_{j}}^{\left(z\right)}$ is the life expectancy computed by applying the KM estimator to the standard data *z*.
- *Estimation step*. The KM estimator is applied to ${\hat{w}}^{\left(0\right)}$ to produce the survival function estimate ${\hat{S}}^{\left(0\right)}\left(t\right)$. In case of incomplete death-observations, the distribution is completed by posing ${\hat{S}}^{\left(0\right)}\left(t_{\max}\right)=0$.
- *Expectation step*. Using ${\hat{w}}^{\left(0\right)}$, the *m* future life expectancy ${\hat{e}}_{\theta_{j}}^{\left(0\right)}$ are computed as in (3). The corresponding time points ${\hat{\tau }}_{j}^{\left(0\right)}$ are then replaced by ${\hat{\tau }}_{j}^{\left(1\right)}=\theta_{j}+{\hat{e}}_{\theta_{j}}^{\left(0\right)}$. One then obtains the new artificial complete data set:
  $${\hat{w}}^{\left(1\right)}=\left\{(t_{i},d_{i}),\;i=1,\cdots ,n\right\}\cup \left\{({\hat{\tau }}_{j}^{\left(1\right)},1),\;j=1,\cdots ,m\right\}\;.$$
- The estimation and the expectation steps are repeated, producing at the *k*-th stage a new complete data set ${\hat{w}}^{\left(k\right)}$, provided by the expectations $\left\{{\hat{e}}_{\theta_{j}}^{\left(k\right)},\;j=1,\cdots ,m\right\}$. The iterations stop when a specified *convergence criterion* is fulfilled. A natural criterion is:
  (6) $$\max_{1\leq j\leq m}\left\{\left|{\hat{e}}_{\theta_{j}}^{\left(k+1\right)}-{\hat{e}}_{\theta_{j}}^{\left(k\right)}\right|\right\}<\varepsilon \;,$$
  for a suitable specified *tolerance level* ε>0 (this choice will be left as an option for the user). If condition (6) is not satisfied after a fixed maximum number of iterations (which will also be chosen as a user option), the convergence is considered failed.

If the convergence criterion is met, the final values of the *m* life expectancy provide estimates which we will denote by ${\hat{e}}_{\theta_{j}}$. The corresponding estimated lifetimes are ${\hat{\tau }}_{\theta_{j}}=\theta_{j}+{\hat{e}}_{\theta_{j}}$ and the estimated whole data set is:

(7) $$\hat{w}=\left\{z_{i}=\left(t_{i},d_{i}\right),\;i=1,\cdots ,n\right\}\cup \left\{{\hat{z}}_{j}^{\prime }=\left({\hat{\tau }}_{j},1\right),\;j=1,\cdots ,m\right\}\;.$$

This iterative procedure can be seen in the class of the well-known *Expectation-Maximization* (EM) algorithms, since the estimation step can be interpreted as a maximization, given that the KM approach provides a maximum likelihood estimator. In this class of algorithms the expectation step is often referred to as *mean-imputation*, hence we will call our iterative procedure *Covid-Death Mean-Imputation* (CoDMI) algorithm.

**Remark** **4.** *(i) Usually EM algorithms, and the concept of imputation, refer to procedures aimed to filling-in missing data. What we are dealing with here is data observed to a limited extent, rather than completely missing. Therefore, in this application the imputation corresponds rather to a replacement(of the observed time points $\theta_{j}$ by the estimated time points $\tau_{j}$). Our method is, however, in the spirit of the fake-data principle, as illustrated by Efron and Hastie* [15]*, pp. 148–149.*

*(ii) It should be noted that the idea of estimating the virtual lifetimes $\tau_{j}$ as the expectation $E(T_{\theta_{j}})$ implies a further more subtle assumption. Let DoC_j_ be the event: “Patient j died of COVID-19 at time $\theta_{j}$” and RoC_j_: “Patient j became ill with COVID-19 but recovered at time $\theta_{j}$”. Using notation introduced by Pearl in causal analysis (e.g.,* [16])*, we are assuming for this patient that:*

$$E\left(T_{\theta_{j}}|do\left({DoC}_{j}=0\right)\right)=E\left(T_{\theta_{j}}|{DoC}_{j}=0\right)\;,$$

*where do(A) is the intervention operator on event A. This means that we are assuming that the event RoC_j_, which is not excluded by DoC_j_ = 0, does not change the probability distribution of $T_{\theta_{j}}$. This is clearly a simplifying assumption that makes our counterfactual problem easy to solve. In a more rigorous analysis, the effect of events as RoC_j_ should be also taken into account* [1]*. We refrain to do this here, since such an analysis would take us out of the KM survival framework.*

### 3.2. The Convergence Issue

In general, CoDMI is not guaranteed to converge. If we make the classical binomial assumptions, we can derive the KM likelihood as a function of the hazard rates $h_{i}$. Running the algorithm, we find it is possible that different parameter sets, then different sets of ${\hat{e}}_{\theta_{j}}$ estimates, correspond to the same likelihood value. This should indicate an issue in parameter identifiability. However the classical KM likelihood is defined for fixed time points, while the estimates ${\hat{e}}_{\theta_{j}}$ change at each step in our algorithm. Thus, the identifiability problem should be more properly studied referring to a likelihood function which includes the event times in the parameters as well.

**Remark** **5.** *A similar problem of iterated estimates for the KM product-limit estimator, but with fixed time points hence without parameter identifiability issues, was studied by Efron* [14]*. He proved in this case that, provided that the probability distribution is complete, the solution of the convergence problem exists and is unique. The previously mentioned self-consistency refers precisely to this property.*

However, in order to manage the convergence problem, even based on the results of the simulation exercise presented in Section 5, it is worth considering the following three types of situation.

(1) *Finite time convergence*. The difference between two successive estimates becomes zero after a finite number of iterations.
(2) *Asymptotic convergence*. The difference between two successive estimates tends to zero asymptotically.
(3) *Cyclicity*. After a certain number of iterations, cycles of the estimated values are established which tend to repeat themselves indefinitely, so that the minimum difference between two successive estimates remains greater than zero. In this case, if this minimal difference is less than the tolerance ε, the corresponding estimate can be accepted (this is actually referred to by the term “tolerance”). It often happens that small changes in some of the $\theta_{j}$ values are sufficient to get out of cyclicity cases. Therefore, some fudging of these data could be used to obtain acceptable solutions when the minimum improvement is out of tolerance.

As shown in the simulation study in Section 5, cases of non-convergence are not very frequent, and many of these can be circumvented by milding the convergence criterion (6) and fudging the COVID-19 data a little, if necessary. In general, the results are found to be sensitive to the initial values ${\hat{\tau }}_{j}^{\left(0\right)}$. In cases of convergence this is not a problem since different solutions, but within the chosen tolerance criterion, are equivalent from a practical point of view. In some cases of non-convergence, on the other hand, it is possible to skip to convergence cases by changing the initial values.

### 3.3. Assumptions Underlying CoDMI

The iterative procedure described in Section 3.1 can probably be easily justified by intuitive reasoning. However, also to give internal consistency to the simulation procedure presented in Section 5, it is convenient to better specify the assumptions underlying the CoDMI algorithm. A preliminary remark is important to be made. In our framework, the “true” probability distribution of the random variable $T_{0}$ is the best-fitting distribution in the KM sense, i.e., the distribution identified by applying the maximum likelihood product-limit estimator to existing data. Without appropriate additional assumptions (e.g., specifying an analytic form of the hazard function) this distribution is completely non-parametric and there is no other way to identify it than by specifying the data as well as the estimator used (the product-limit estimator, in fact). One could say, data provide information to the estimator, and the estimator provides probabilistic structure to data. Having remarked upon this, the basic assumption underlying CoDMI algorithm outlined in the following section. When COVID-19 deaths are present in the study sample, there is an extended underlying data structure composed of the *n* observed lifetimes $t_{i}$ (ending with a DoD or a Cen) and by the *m* partially observed lifetimes $\tau_{j}$ (virtually ending, if we assume $\delta_{j}=1$, with a DoD). The corresponding probability distribution is the best-fitting distribution specified by this extended data, i.e., by applying the KM estimator to the data set $w=z\cup z^{\prime }$. We will keep have this property in mind when we generate the simulated scenarios on which to measure the algorithm’s predictive performance.

### 3.4. Adjusting for the Assumption $\delta_{j}\equiv 1$

Relaxing the assumption that patients eliminated by a DoC event would have died of disease without this event is not an easy task. The prediction regarding the status operators $\delta_{j}$ increases the forecasting problem by one dimension and requires a reliable predictive model, which is currently not available to us. We are therefore content to propose an *adjustment for censoring* of the response of CoDMI algorithm which should mitigate the possible bias produced by the assumption $\delta_{j}\equiv 1$. If the algorithm met the convergence criterion, the final data set is given by (7). We then consider the modified data set:

(8) $${\hat{w}}^{\left(R\right)}=\left\{(t_{i},1-d_{i}),\;i=1,\cdots ,n\right\}\cup \left\{({\hat{\tau }}_{j},0),\;j=1,\cdots ,m\right\}\;,$$

where both the observed and the estimated virtual lifetimes are kept the same, while all the status indicators are reversed. Running the KM estimator on the set ${\hat{w}}^{\left(R\right)}$, one obtains the so-called *reverse Kaplan–Meier survival curve* ${\hat{S}}^{\left(R\right)}\left(t\right)$, which refers to Cen instead of DoD endpoints, and provides the new conditional expectations ${\hat{\tau }}_{j}^{\left(R\right)}$, given θ, of the virtual lifetimes. We then choose to derive the adjusted estimates ${\hat{\tau }}_{j}^{*}$, for j=1,⋯,m, as:

(9) $${\hat{\tau }}_{j}^{*}=\left\{\begin{array}{c} {\hat{\tau }}_{j}\;\;\;\;\mathrm{if}\;\alpha \left(\theta_{j}\right)\geq 0.5\;,\\ {\hat{\tau }}_{j}^{\left(R\right)}\;\mathrm{if}\;\alpha \left(\theta_{j}\right)<0.5\;, \end{array}\right.$$

where α(t) is the probability that an event observed at time *t* is a DoD (as opposed to a Cen). In order to estimate these non-censoring probabilities, the standard observations $\left\{z_{i}=\left(t_{i},d_{i}\right)\right\}$ are represented on a time grid spanning the time interval $\left[0,t_{\max}\right]$ with cells l=1,2,⋯,G, and a parametric hazard rate function ${\hat{h}}_{l}$ is fitted on this grid. The same procedure is then applied to the “reverse observations” $\left\{z_{i}^{\left(R\right)}=\left(t_{i},1-d_{i}\right)\right\}$ and the corresponding hazard rate function ${\hat{h}}_{l}^{\left(R\right)}$ is then derived. The probability estimates are then computed as $\hat{\alpha }\left(t\right)={\hat{h}}_{l(t)}/\left[{\hat{h}}_{l(t)}+{\hat{h}}_{l(t)}^{\left(R\right)}\right]$, where l(t) is the cell containing the time point *t*. Examples of estimated $\hat{\alpha }\left(t\right)$ functions are provided in the next section.

The above procedure is fairly ad hoc and the indications provided do not necessarily have to be accepted. It may be the case that the user of the procedure has a personal opinion, based on external information, on the value of (some of) the virtual status operator $\delta_{j}$. In this situation the coefficients $\alpha (\theta_{j})$ in (9) could be assigned or modified by the user on the basis of this expert judgment.

### 3.5. An Extended Greenwood’s Formula

The virtual lifetime expectations ${\hat{\tau }}_{j}$ provided by CoDMI and included in the mean-imputed data $\hat{w}$ are point estimates which allow these data to be applied to any statistical tool available for survival analysis. However, replacing an observed value with a point estimate, even an unbiased one, increases the variance of the survival estimates, since the mean-imputed data convey their own estimation error. Usually the standard deviation of the KM survival function estimate is computed using Greenwood’s formula. On the standard data, using the same notation in (1), this can be written as:

(10) $$s.d.\left(\hat{S}\left(t\right)\right)=\hat{S}\left(t\right)\;{\left[\sum_{i:t_{\left(i\right)}\leq t}\frac{h_{\left(i\right)}}{1-h_{\left(i\right)}}\;\frac{1}{R(t_{\left(i\right)})}\right]}^{1/2},\;\mathrm{with}\;h_{\left(i\right)}=\frac{d_{\left(i\right)}}{R(t_{\left(i\right)})}\;,$$

where the summand is set to 0 if $h_{\left(i\right)}=1$. We provide an extension of this formula in order to include the variance component due to the estimated time points ${\hat{\tau }}_{j}$.

We start by the CoDMI output, eventually with the adjustment for censoring:

$$\hat{w}=\left\{(t_{i},d_{i}),\;i=1,\cdots ,n\right\}\cup \left\{({\hat{\tau }}_{j}^{*},\delta_{j}),\;j=1,\cdots ,m\right\}$$

where the ${\hat{\tau }}_{j}^{*}$ are derived by (9) and the indicators $\delta_{j}$ can be equal to 0 or 1. We represent the $\hat{w}$ data set in the alternative form:

$$\hat{y}=\left\{(t_{i}^{\prime },d_{i}^{\prime },\delta_{i}^{\prime }),\;i=1,\cdots ,n+m\right\}\;,$$

where:

- $t_{i}^{\prime }=t_{i}$ or ${\hat{\tau }}_{j}^{*}$ are the observed or estimated survival times *ordered* by increasing value (the usual conventions on tied values apply);
- $d_{i}^{\prime }=0$ if $t_{i}^{\prime }$ corresponds to a Cen and 1 otherwise;
- $\delta_{i}^{\prime }=1$ if $t_{i}^{\prime }$ corresponds to a DoC and 0 otherwise.

Since the time points $t_{i}^{\prime }$ are assumed to be ordered, we simplify the exposition in this section by using the subscript *i* instead of (i) (and $R_{i}$ instead of $R(t_{\left(i\right)})$). We then consider both the “direct” probability distribution $\left\{q_{i},\;i=1,\cdots ,n+m\right\}$ and the reverse one $\left\{q_{i}^{\left(R\right)},\;i=1,\cdots ,n+m\right\}$, both taken from the CoDMI output, and from these we derive the *m* direct and the *m* reverse truncated distributions:

$$q_{i,j}=q_{i}\;\frac{1_{\left\{t_{i}>\theta_{j}\right\}}}{\sum_{k:t_{k}>\theta_{j}}q_{k}},\;q_{i,j}^{\left(R\right)}=q_{i}^{\left(R\right)}\;\frac{1_{\left\{t_{i}>\theta_{j}\right\}}}{\sum_{k:t_{k}>\theta_{j}}q_{k}^{\left(R\right)}},\;i=1,\cdots ,n+m\;,\;j=1,\cdots ,m\;.$$

These distributions are defined, with null values, also for $t_{i}^{\prime }\leq \theta_{j}$. Finally, we compute the *total probabilities*:

(11) $$Q_{i}=\sum_{j=1}^{m}q_{i,j}^{*}\;,\;with\;q_{i,j}^{*}=\delta_{j}\;q_{i,j}+\left(1-\delta_{j}\right)\;q_{i,j}^{\left(R\right)}\;,\;i=1,\cdots ,n+m\;,$$

and define $Q_{i}^{\left(2\right)}=\sum_{j=1}^{m}{\left(q_{i,j}^{*}\right)}^{2},\;i=1,\cdots ,n+m$. Observe that $\sum_{j=1}^{m}Q_{i}=m$.

With these definitions, we propose the following correction of Greenwood’s formula:

(12) $$s.d.\left(\hat{S}\left(t\right)\right)=\hat{S}\left(t\right)\;{\left[\sum_{i:t_{i}^{\prime }\leq t}\;\left(\frac{{\bar{h}}_{i}}{1-{\bar{h}}_{i}}\;\frac{1}{{\bar{R}}_{i}}+\frac{1}{{\left(1-{\bar{h}}_{i}\right)}^{2}}\;\frac{{\bar{R}}_{i}-1}{{\bar{R}}_{i}}\;\frac{Q_{i}-Q_{i}^{\left(2\right)}}{{\bar{R}}_{i}^{2}}\right)\right]}^{1/2}\;,$$

where the hazard rates ${\bar{h}}_{i}$ are specified as:

$${\bar{h}}_{i}=\frac{d_{i}^{\prime }\;\nu_{i}}{{\bar{R}}_{i}},\;with\;\nu_{i}=\left(1-\delta_{i}^{\prime }\right)+Q_{i},\;i=1,2,\cdots ,n+m\;,$$

and the number of subjects at risk is computed as:

$${\bar{R}}_{i}=\left\{\begin{array}{c} n+m\;\mathrm{for}\;i=1,\\ {\bar{R}}_{i-1}-\left(1+\left(\nu_{i}-1\right)\;d_{i}^{\prime }\right)\;\mathrm{for}\;i=2,3,\cdots ,n+m. \end{array}\right.$$

The basic idea underlying this formula is that the *m* COVID-19 deaths are distributed as “fractional deaths” $Q_{i}=\sum_{j}q_{i,j}^{*}$ over all the uncensored time points (both DoD and DoC), and the hazard rate at time $t_{i}^{\prime }$ has a random component with mean $Q_{i}/{\bar{R}}_{i}$ and variance $\left(Q_{i}-Q_{i}^{\left(2\right)}\right)/{\bar{R}}_{i}^{2}$. The details of the derivation of Formula (12) are provided in Appendix A. Using (12), the approximate 95% confidence intervals can be computed by:

(13) $$\log\left(\hat{S}\left(t\right)\right)\pm 1.96\;\frac{s.d.\left(\hat{S}\left(t\right)\right)}{\hat{S}\left(t\right)}\;.$$

## 4. Examples of Application to Real Survival Data

### 4.1. Application to COVID-19 Extended NCOG Data

To illustrate the effects of our mean-imputation adjustments, we start by considering some *real* survival data well referenced in the literature and apply CoDMI algorithm to these data after the addition of some *artificial* COVID-19 deaths. This is carried out because, currently, sufficiently rich real datasets containing both cancer-death and Covid-death events are hardly available. To this aim, we chose, as the real reference data, the head/neck cancer data of the NCOG (North Carolina Oncology Group) study, which was used to illustrate the KM approach in the book by Efron and Hastie, Section 9.2 [15]. We considered data from the two arms, A and B, separately.

#### 4.1.1. Arm A of NCOG Data

Survival times (in days) from Arm A in the first panel of Table 9.2 [15] are reported in Table 1.

**Figure 1:**
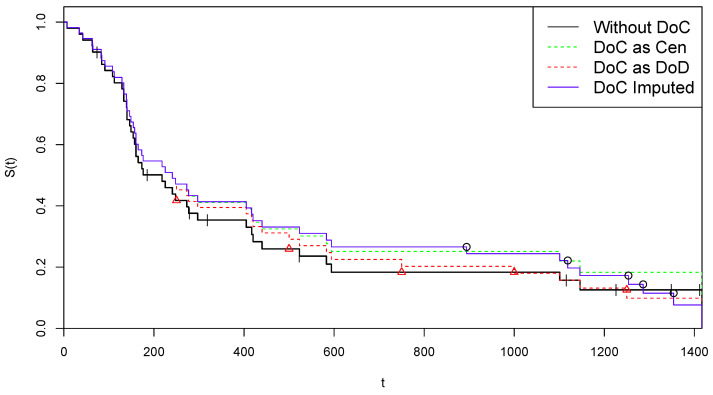
Kaplan–Meier curves for alternative treatments of COVID-19 deaths—Arm A.

To save space, data is presented, as in the book, in compact form, with the + sign representing censoring. The conversion of these data into the form of a two-component vector $z=\{\left(z_{i},d_{i}\right),\;i=1,2,\cdots ,n\}$ is immediate. There are n=51 patients, with 43 DoD events and 8 Cen events. The final time point is 1417 days after the beginning of the study, and a DoD is observed on that date. Therefore we are in a complete death-observations case, with $t_{max}=t_{\max}^{\left(D\right)}=1417$. The corresponding KM estimate of the survival function $\hat{S}\left(t\right)$ is illustrated by the black line in Figure 1.

To illustrate the application of CoDMI algorithm, we add to these data an artificial group of *m* Covid death observations, i.e., *m* DoC events assumed being observed at the time points $\theta =\{\theta_{j},\;j=1,2,\cdots ,m\}$. We chose m=5 (roughly 10% of *n*) DoC events, on 5 time points roughly equally spaced in $\left(0,t_{\max}\right)$:

(14) θ={250,500,750,1000,1250}.

Since the observation set x=z∪θ has been specified, we have to choose the virtual lifetimes ${\hat{\tau }}_{j}^{\left(0\right)}$ in the data set ${\hat{w}}^{\left(0\right)}$ which is used to initialize CoDMI algorithm. If, for example, we choose the option to set ${\hat{\tau }}_{j}^{\left(0\right)}\equiv \theta_{j}$, then we have ${\hat{w}}^{\left(0\right)}=z\cup {\hat{z}}^{\prime (0)}$, with:

$${\hat{z}}^{\prime (0)}=\left\{\left(250,1\right),\;\left(500,1\right),\;\left(750,1\right),\;\left(1000,1\right),\;\left(1250,1\right)\right\}\;.$$

We run CoDMI algorithm with this initialization and ε=0.1. The procedure converged after 10 iterations, providing the following estimates for the lifetimes $\left\{{\hat{\tau }}_{j},\;j=1,\cdots ,5\right\}$:

(15) {894.32,1118.85,1253.58,1286.24,1354.00}.

The corresponding COVID-19 data:

(16) $${\hat{z}}^{\prime }=\left\{\left(894.32,1\right),\;\left(1118.85,1\right)\;\left(1253.58,1\right),\;\left(1286.24,1\right),\;\left(1354.00,1\right)\right\}\;,$$

are then used as mean-imputed data to obtain the final complete data set $\hat{w}$ in (7). As one can observe, the expectation Formula (3) provides non-integer values, which is not a problem since the survival function provided by the KM estimator is defined on the real axis.

**Remark** **6.** *A tolerance of 0.1 already provides overabundant precision for our applications. However, in order to stress the algorithm, we also tried with $\varepsilon =10^{-8}$ and $\varepsilon =10^{-18}$, obtaining convergence after 33 and 51 iterations, respectively. This seems to be a case of asymptotic convergence.*

The survival curve provided by the KM estimator applied to the completed data $\hat{w}$ (“DoC Imputed”) is illustrated in blue color in Figure 1, where it can be compared with the original survival estimate based on the *z* data (“Without DoC”, black color). For further comparisons, we also present the survival KM curves estimated by the two naïve strategies, comprising a classification of all DoC events as Cen, i.e., ${\hat{\tau }}_{j}\equiv \theta_{j}$ and $\delta_{j}\equiv 0$ (“DoC as Cen”, green color), or all DoC events as DoD, i.e., ${\hat{\tau }}_{j}\equiv \theta_{j}$ and $\delta_{j}\equiv 1$ (“DoC as DoD”, red). In the figure, the “critical” time points are reported by indicating the 14 Cen points by tiks and the 5 $\theta_{j}$ points by red triangles on the black curve, while the 5 ${\hat{\tau }}_{j}$ points are indicated by circles on the blue line (where, obviously, each circle corresponds to a jump).

We finally illustrate the application of the adjustment for censoring presented in Section 3.4. After deriving from $\hat{w}$ the modified data set ${\hat{w}}^{\left(R\right)}$ in (8), we apply the KM estimator to these data, obtaining the following alternative lifetimes $\left\{{\hat{\tau }}_{j}^{\left(R\right)},\;j=1,\cdots ,5\right\}$:

(17) {1207.49,1296.23,1347.78,1347.78,1398.13}.

**Figure 2:**
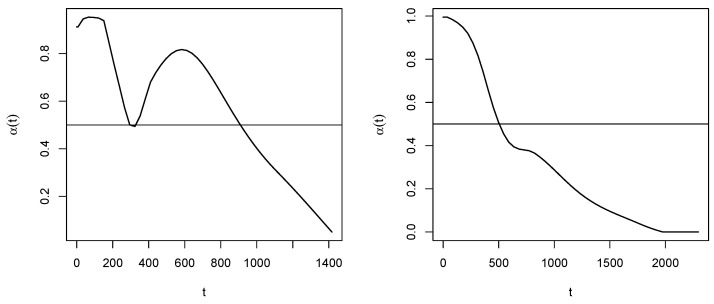
Non-censoring probability curves for Arm A (**left**) and Arm B (**right**).

In Figure 2, on the left it is illustrated the probability curve α(t) estimated as specified in Section 3.4. By this function, one obtains:

$$\alpha \left(\theta_{1}\right)=0.623,\;\alpha \left(\theta_{2}\right)=0.781,\;\alpha \left(\theta_{3}\right)=0.699,\;\alpha \left(\theta_{4}\right)=0.402,\;\alpha \left(\theta_{5}\right)=0.193\;.$$

Therefore, the procedure suggests to consider the last two time points as (potentially) censored, then estimated as in (17). The data set ${\hat{z}}^{\prime }$ in (16) is then modified as:

$${\hat{z}}^{\prime }=\left\{\left(894.32,1\right),\;\left(1118.85,1\right)\;\left(1253.58,1\right),\;\left(1347.78,0\right),\;\left(1398.13,0\right)\right\}\;.$$

These suggestions, however, are purely indicative and can be rejected or changed based on expert opinion.

**Figure 3:**
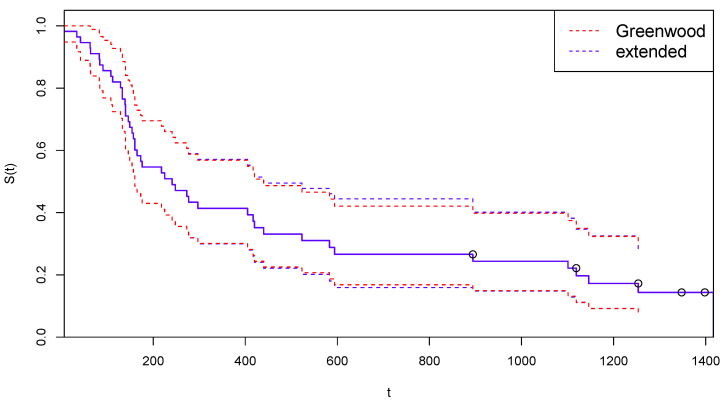
Kaplan–Meier curves estimated by CoDMI with adjustment for censoring and related confidence intervals—Arm A.

In Figure 3, the survival function estimated after the suggested adjustment for censoring is reported, together with the 95% confidence limits computed with the traditional Greenwood’s formula (red dotted lines) and with the extended Formula (12) (blue dashed lines).

#### 4.1.2. Arm B of NCOG Data

In Table 2, we report censored survival times (in days) from Arm B in the second panel of Table 9.2 [15].

Furthermore, in this case, we refrain, for reasons of space, to present data converted into *z* form. Data are heavily censored in this arm, having n=45 patients, with 14 Cen events, which are mainly distributed among the largest time points. Moreover, we are in a case of incomplete death-observations, since the final time point $t_{\max}=2297$ is a Cen point. The last time point with a DoD event observed is $t_{\max}^{\left(D\right)}=1776$ and 4 Cen events are observed thereafter. The final level of the survival curve provided by the KM estimator is $\hat{S}\left(t_{\max}^{\left(D\right)}\right)=22.99\%$ and we choose to allocate this probability mass entirely on the final Cen point 2297. For the artificial data on COVID-19 deaths, also in this case we choose *m* roughly 10%n and assume equally spaced DoC events in the interval (0,2297). That is we assume n=5 with $\theta_{j}$ values in the set:

(18) θ={400,800,1200,1600,2000}.

The last time point in θ is after the last observed DoD time point (1776). As in the previous case, the initial data set ${\hat{z}}^{\prime }$ is derived by setting $\delta_{j}\equiv 1$, and the complete data set ${\hat{w}}^{\left(0\right)}=z\cup {\hat{z}}^{\prime }$ is used to initialize CoDMI algorithm. The algorithm, run again with ε=0.1, converged after 12 iterations (convergence was met after 49 iterations for $\varepsilon =10^{-8}$ and 78 iterations for $\varepsilon =10^{-18}$), providing the following estimates for the adjusted lifetimes $\left\{{\hat{\tau }}_{j},\;j=1,\cdots ,5\right\}$:

{1654.63,1934.24,2004.07,2041.32,2148.59}.

**Figure 4:**
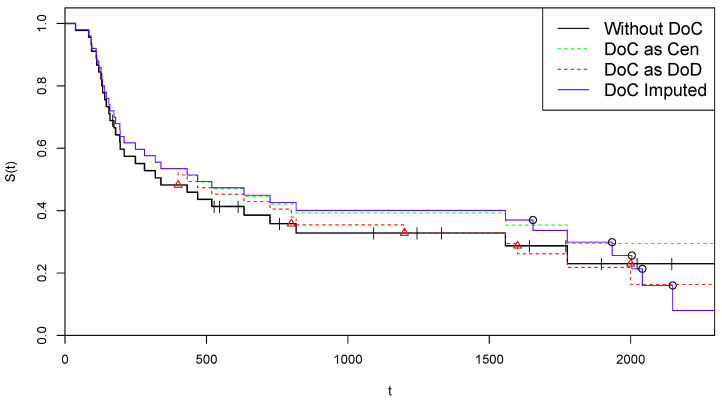
Kaplan–Meier curves for alternative treatments of COVID-19 deaths—Arm B.

In Figure 4, we replicate the illustrations of Figure 1 on these data. As concerning the adjustment for censoring, from the estimated probability curve reported in Figure 2 on the right we obtain:

$$\alpha \left(\theta_{1}\right)=0.667,\;\alpha \left(\theta_{2}\right)=0.371,\;\alpha \left(\theta_{3}\right)=0.192,\;\alpha \left(\theta_{4}\right)=0.074,\;\alpha \left(\theta_{5}\right)=0.0002\;.$$

Therefore, in this case, the procedure suggests to consider the last four time points as censored. Using the criterion in (17), the final data set is obtained:

$${\hat{z}}^{\prime }=\left\{\left(1654.63,1\right),\;\left(1922.76,0\right),\;\left(1978.15,0\right),\;\left(2084.32,0\right),\;\left(2201.93,0\right)\right\}\;.$$

**Figure 5:**
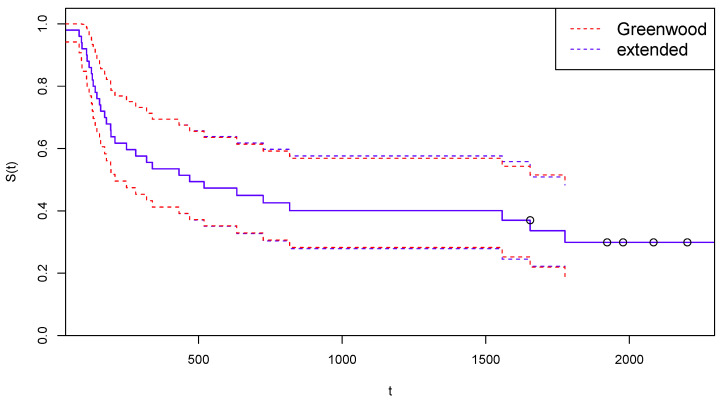
Kaplan–Meier curves estimated by CoDMI with adjustment for censoring and related confidence intervals—Arm B.

Figure 5 is the analogous for Arm B of Figure 3.

## 5. A Simulation Study

In order to test the ability of CoDMI to correctly estimate the expected life-shortening (or the corresponding virtual lifetime) due to DoC events in a study population, we generate many scenarios each containing simulated data. These pseudo-data include a $\tilde{z}$ data set of standard observations and a ${\tilde{\tau }}^{\left(0\right)}$ data set of (preliminary) virtual lifetimes. By randomly censoring the time variables in ${\tilde{\tau }}^{\left(0\right)}$ a corresponding set $\tilde{\theta }$ of DoC time points is derived. In order to equip these pseudo-data with a probabilistic structure consistent with CoDMI assumptions, a KM best-fitting distribution is derived by applying the product-limit estimator to $\tilde{z}\cup {\tilde{\tau }}^{\left(0\right)}$. The “true” virtual lifetimes $\tilde{\tau }$ are then derived by conditional sampling, given $\tilde{\theta }$, from this distribution. Running CoDMI algorithm on the pseudo-observations $\tilde{x}=\tilde{z}\cup \tilde{\theta }$, the estimated virtual lifetimes $\hat{\tau }$ are obtained and the quality of the estimator is measured by computing the average, over all scenarios, of the prediction errors $\tilde{\tau }-\hat{\tau }$.

### 5.1. Details of the Simulation Process

The details of each scenario simulation are as follows:

1. *Simulation of standard survival data $\tilde{z}$.* The simulated standard (i.e., non-Covid) survival data $\tilde{z}$ is generated in each scenario starting from the same set of real data $z=\{\left(t_{i},d_{i}\right),i=1,2,\cdots n\}$, spanning the time interval $\left[0,t_{\max}\right]$. The set $\tilde{z}$ is generated by drawing with replacement $n_{sim}$ pairs $\left({\tilde{t}}_{i},{\tilde{d}}_{i}\right)$ from the *n* real-life pairs $\left(t_{i},d_{i}\right)$, maintaining the proportion between DoD and Cen events in *z*. Let us denote by ${\tilde{t}}_{\max}^{\left(D\right)}$ the largest uncensored time point in $\tilde{z}$.
  *Remark*. It should be noted that many tied values can be generated in this step, especially if $n_{\mathrm{sim}}\gg n$. Moreover, ${\tilde{t}}_{\max}^{\left(D\right)}$ could result to be censored (a case of incomplete death observations) even if the death observations are complete in the original data. It is easy to guess that generating many scenarios in this way can produce a number of “extreme” pseudo-data $\tilde{z}$. This is useful, however, for testing the algorithm even in unrealistic situations. Most cases of failed convergence correspond to extreme situations.
2. *Simulation of DoC time points $\tilde{\theta }$.* In order to simulate a number $m_{\mathrm{sim}}$ of COVID-19 deaths, the time points ${\tilde{\tau }}_{j}^{\left(0\right)},\;j=1,2,\cdots ,m_{\mathrm{sim}},$ are generated by drawings with replacement from the $t_{i}$ points in real data *z*, satisfying the conditions $d_{i}=1$ and $t_{i}\leq {\tilde{t}}_{\max}^{\left(D\right)}$. These time points are interpreted as temporary virtual lifetimes and are first used to generate the DoC time points ${\tilde{\theta }}_{j}$. A number $m_{\mathrm{sim}}$ of independent drawings ${\tilde{u}}_{j}$ from a uniform (0, 1) distribution are performed, and the corresponding DoC time points are obtained as ${\tilde{\theta }}_{j}={\tilde{u}}_{j}\cdot \;{\tilde{\tau }}_{j}^{\left(0\right)}$. Therefore, for all *j* one has $0<{\tilde{\theta }}_{j}<{\tilde{\tau }}_{j}^{\left(0\right)}\leq {\tilde{t}}_{\max}^{\left(D\right)}$, with ${\tilde{\theta }}_{j}$ taking equally probable values in $\left(0,{\tilde{\tau }}_{j}^{\left(0\right)}\right)$.
  *Remark*. The use of a uniform distribution is obviously questionable, and more “informative” distribution could be suggested. For example, a beta distribution with first parameter greater than 1 and second parameter lower than 1 may be preferable, as it makes more probable values of ${\tilde{\theta }}_{j}$ closer to ${\tilde{\tau }}_{j}^{\left(0\right)}$. However, the form of this distribution is irrelevant to our purposes: we are interested in observing how CoDMI is able to capture the simulated virtual lifetimes, independently of how they are generated.
3. *Simulation of virtual lifetimes ${\tilde{\tau }}_{j}$.* The temporary lifetimes ${\tilde{\tau }}_{j}^{\left(0\right)}$ (and the data set $\tilde{z}$) cannot be directly used to test CoDMI algorithm, since their probabilistic structure is indeterminate and, in any case, we have too few (pseudo-)observations. In order to introduce a probabilistic structure consistent with CoDMI assumptions, we first run the KM estimator on the data set ${\tilde{w}}^{\left(0\right)}=\tilde{z}\cup \left\{\left({\tilde{\tau }}_{j}^{\left(0\right)},1\right)\right\}$, thus obtaining the corresponding death probability distribution $\left\{{\tilde{q}}_{i}^{\left(0\right)},\;i=1,2,\cdots n_{\mathrm{sim}}+m_{\mathrm{sim}}\right\}$. The virtual lifetimes ${\tilde{\tau }}_{j}^{\left(1\right)},\;j=1,2,\cdots ,m_{\mathrm{sim}},$ are then obtained by computing the conditional expectations $E(T_{{\tilde{\theta }}_{j}})$ by this distribution. However, this is not yet fully consistent with CoDMI assumptions, since, as discussed in Section 3.3, the appropriate distribution is the KM best-fitting distribution specified on the extended data, i.e., data including the virtual lifetimes themselves. To obtain this result we should repeat the previous step, i.e., running the product-limit estimator on the new data set ${\tilde{w}}^{\left(1\right)}=\tilde{z}\cup \left\{\left({\tilde{\tau }}_{j}^{\left(1\right)},1\right)\right\}$, thus producing the new distribution $\left\{{\tilde{q}}_{i}^{\left(1\right)},\;i=1,2,\cdots n_{\mathrm{sim}}+m_{\mathrm{sim}}\right\}$ and then simulating $m_{sim}$ new time points ${\tilde{\tau }}_{j}^{\left(2\right)}$ by taking the conditional expectation on this distribution. In principle, this step should be iterated similarly to what is completed in the CoDMI algorithm. To avoid convergence problems, however, we prefer to limit the number of iterations to a fixed (low) value $n_{iter}$, thereby implicitly accepting a certain level of bias in the estimations. After these $n_{\mathrm{iter}}$ iterations has been made, the final data set ${\tilde{w}}^{\left(n_{\mathrm{iter}}\right)}=\tilde{z}\cup \left\{\left({\tilde{\tau }}_{j}^{\left(n_{\mathrm{iter}}\right)},1\right)\right\}$ is obtained. Running the KM estimator on these data again, the final distribution $\left\{{\tilde{q}}_{i}^{\left(n_{\mathrm{iter}}\right)},\;i=1,2,\cdots n_{\mathrm{sim}}+m_{\mathrm{sim}}\right\}$ is obtained and the definitive time points ${\tilde{\tau }}_{j}$, with the corresponding ${\tilde{e}}_{j}={\tilde{\tau }}_{j}-\theta_{j}$, are computed *by conditional sampling*, given ${\tilde{\theta }}_{j}$, i.e., simulating from the truncated distribution $\left\{{\tilde{q}}_{i}^{\left(n_{iter}\right)},\;i:t_{i}>\theta_{j}\right\}$ (after normalization). These sampled values are taken as the *true* values of virtual lifetimes and life expectancy, respectively, which should be estimated by CoDMI using only the information $\tilde{z}\cup \tilde{\theta }$.
4. *Application of CoDMI and naïve estimators.* CoDMI algorithm is applied to the simulated data:
  $$\tilde{w}=\left\{{\tilde{z}}_{i}=\left({\tilde{t}}_{i},{\tilde{d}}_{i}\right),\;i=1,\cdots ,n_{\mathrm{sim}}\right\}\cup \left\{{\tilde{z}}_{j}^{\prime }=\left({\tilde{\theta }}_{j},1\right),\;j=1,\cdots ,m_{\mathrm{sim}}\right\}\;,$$
  with ${\tilde{z}}_{i}$ obtained in step 1 and ${\tilde{\theta }}_{j}$ in step 2. Provided that the algorithm converges, we obtain the $m_{\mathrm{sim}}$ estimated virtual lifetimes ${\hat{\tau }}_{j}$ and the estimated life expectancy ${\hat{e}}_{j}$.
  To allow comparison, we also derive in this step the predictions of the two naïve “estimators” which are obtained by applying the KM estimator to the simulated data $\tilde{w}$, modified by posing, for all *j*, ${\tilde{\tau }}_{j}={\tilde{\theta }}_{j}$ and $\delta_{j}=1$ (“DoC as DoD”) or $\delta_{j}=0$ (“DoC as Cen”).

### 5.2. Valuation of the Predictive Performances

In the simulation exercise, a large number *N* of scenarios are generated. This provides, for $j=1,2,\cdots ,m_{\mathrm{sim}}$ and k=1,2,⋯,N, the $N\cdot m_{\mathrm{sim}}$ CoDMI estimates ${\hat{e}}_{j}^{\left(k\right)}$ (from step 4) and the $N\cdot m_{\mathrm{sim}}$ true realizations ${\tilde{e}}_{j}^{\left(k\right)}$ (from step 3). Then we can compute the prediction errors:

$$\Delta_{j}^{\left(k\right)}={\tilde{e}}_{j}^{\left(k\right)}-{\hat{e}}_{j}^{\left(k\right)},\;j=1,2,\cdots ,m_{\mathrm{sim}},\;k=1,2,\cdots ,N\;,$$

and the average errors:

$${\bar{\Delta }}_{j}=\frac{1}{N}\sum_{k=1}^{N}\Delta_{j}^{\left(k\right)}\;,\;\;j=1,2,\cdots ,m_{\mathrm{sim}}\;,\;\bar{\Delta }=\frac{1}{m_{\mathrm{sim}}}\sum_{j=1}^{m_{\mathrm{sim}}}{\bar{\Delta }}_{j}\;.$$

Positive (negative) values of $\Delta_{j}^{\left(k\right)}$ correspond to under(over)-estimates provided by CoDMI. As usual, we can associate to these average errors the corresponding standard error, i.e., the *standard error of the mean* (s.e.m.). Given the independence assumption, the central limit theorem guarantees, as usual, that the sample means are asymptotically normal. Therefore, the corresponding s.e.m. is inversely proportional to $\sqrt{N}$.

The same summary statistics are computed for the prediction errors relative to the two naïve estimators.

### 5.3. Results from Simulation Exercises

Two separate simulation exercises were performed, one using Arm A, the other using Arm B as real-life data. In both the exercises, N=10,000 scenarios were generated, with $n_{\mathrm{sim}}=100$ standard observations (roughly double the real ones) and $m_{\mathrm{sim}}=10$ COVID-19 deaths. A tolerance ε=1 was chosen for the CoDMI algorithm, with a maximum number of allowed iterations ${\mathrm{iter}}_{\max}=100$. The number of iterations for generating the true values was $n_{\mathrm{iter}}=10$ and for all the initializations the option ${\hat{\tau }}_{j}^{\left(0\right)}=\theta_{j}+{\hat{e}}_{\theta_{j}}^{\left(z\right)}$ was chosen. Since in some scenarios CoDMI failed to converge (with the chosen values for ε and ${\mathrm{iter}}_{\max}$), the sample means and the corresponding s.e.m. where computed only on the $N_{c}$ convergence cases.

In Table 3, which is referred to Arm A data, the simulation results are reported for each of the 10 DoC cases. We obtained $N_{c}=9802$ convergence cases out of the 10,000 simulated. In each row, the sample mean of the DoC time points ${\tilde{\theta }}_{j}$, the true life expectancy ${\tilde{e}}_{j}$ and the CoDMI estimated life expectancy ${\hat{e}}_{j}$ are reported in columns 2–4. In columns 5–9, we provide summary statistics of the corresponding prediction errors: the mean error ${\bar{\Delta }}_{j}={\bar{\tilde{e}}}_{j}-{\bar{\hat{e}}}_{j}$, the related s.e.m., the relative mean error ${\bar{\Delta }}_{j}/{\bar{\tilde{e}}}_{j}$ and the minimum and maximum value of ${\bar{\Delta }}_{j}$.

The same results for 10,000 scenarios generated by Arm B data are reported in Table 4.

Table 5 provides the results in Table 3 and Table 4 aggregated over all the DOC events. These overall results are summarized in blok “DoC imputed”. In the bloks, “DoC as DoD” and ”DoC as Cen” the average prediction errors are reported for the two corresponding naïve estimators. The main finding from the simulations is that the CoDMI estimates seem to be essentially unbiased, with a relative prediction error of around 0.5% for both the original data considered. Some more extensive (and time consuming) tests, with $N=10^{5}$ or $N=10^{6}$, have shown a further reduction of the error (as well as, obviously, of the corresponding s.e.m.).

As a final exercise, we used a modified version of the simulation procedure to obtain an assessment of goodness of the adjustment for censoring described in Section 3.4. In the modified simulation, all the $m_{\mathrm{sim}}$ true virtual lifetimes ${\tilde{\tau }}_{j}$ were generated assuming a censoring, instead of a DoC, as the endpoint. Then we set $\delta_{j}\equiv 0$ and in step 3 of Section 5 we generated in all iterations the virtual lifetimes ${\tilde{\tau }}_{j}$ using the truncated *reverse* probability distribution, i.e., the distribution obtained by applying the Kaplan–Meier estimator to the reversed data ${\tilde{w}}^{\left(R\right)}$ (see (8)). Correspondingly with this change in assumption, the estimated values ${\hat{\tau }}_{j}$ in each simulation were obtained by applying the CoDMI algorithm with the final adjustment for censoring, setting at 0 all the probabilities $\alpha (\theta_{j})$ in (9). The overall results from these simulations are summarized in Table 6, which have the same structure as Table 5 and where the results without adjustment are also provided for comparison.

As we can see, the changed assumption on the status of the DoC endpoints provides a large increase of the true life expectancy $\tilde{e}$, but the adjustment for censoring seems to capture quite well this change. Of course, in real life we do not know what the true value of the $\delta_{j}$ is, and we will have to try to choose the suitable ${\hat{\tau }}_{j}$ in (9) based on the $\alpha_{j}$ probabilities and/or using expert judgment.

## 6. Conclusions and Directions for Future Research

In the simulated scenarios, where all the virtual endpoints of COVID-19 cases are assumed to be DoD, the results indicate that CoDMI estimator is roughly unbiased and outperforms alternative estimates obtained by the naïve approaches. In the opposite extreme situation, where all the virtual endpoints of COVID-19 cases are assumed to be censored, the final adjustment for censoring of CoDMI also guarantees unbiasedness, provided that the information on the status of DoC events is assumed to be known. The non-convergence cases can often be circumvented by milding the convergence criterion and/or fudging COVID-19 data a little. Furthermore, changing the initialization of the algorithm can be useful in some cases.

By a natural extension of the binomial assumptions underlying the KM estimator, a version of the classical Greenwood formula can be derived for computing the variance of CoDMI estimates. Equipped with this formula, the CoDMI algorithm is proposed as a complete statistical estimation tool.

As we pointed out in the Introduction, CoDMI algorithm, compared with the cumulative incidence functions method often used to study competing risks, is a pragmatic approach that allows to directly apply all standard statistical tools to “augmented” data. However, it remains important to compare the predictive performance of the two approaches. In our applications, where the competing events are DoD and DoC, we do not yet have sufficiently rich data to test the effectiveness—and possibly the necessity—of an approach based on the cumulative incidence functions, or even to test the possibility of using the two methods in conjunction. Therefore, this topic is left for future research.

Another interesting issue is the convergence of CoDMI algorithm, which is discussed in Section 3.2. A natural way to approach this problem is to study the behavior of the log-likelihood function. However, as we have pointed out, we are not in a fixed time points situation. So it is not a trivial task to explicitly write the updated log-likelihood at each iteration step, because the replacements in each step imply a re-ordering of the time points and consequently a change in the number of items at risk in each death probability estimate. This problem is also left as a future work.

## Figures and Tables

**Table 1:** Censored survival times from Arm A (Chemotherapy) of the NCOG study.

7	34	42	63	64	74+	83	84	91
108	112	129	133	133	139	140	140	146
149	154	157	160	160	165	173	176	185+
218	225	241	248	273	277	279+	297	319+
405	417	420	440	523	523+	583	594	1101
1116+	1146	1226+	1349+	1412+	1417			

**Table 2:** Censored survival times from Arm B (Chemotherapy+Radiation) of the NCOG study.

37	84	92	94	110	112	119	127	130
133	140	146	155	159	169+	173	179	194
195	209	249	281	319	339	432	469	519
528+	547+	613+	633	725	759+	817	1092+	1245+
1331+	1557	1642+	1771+	1776	1897+	2023+	2146+	2297+

**Table 3:** Results by DoC event from N=10,000 simulations ($N_{c}=9802$) generated by Arm A data.

				Summary Statics of ${\bar{\Delta }}_{j}={\bar{\tilde{e}}}_{j}-{\bar{\hat{e}}}_{j}$				
** j **	** ${\bar{\tilde{\theta }}}_{j}$ **	** ${\bar{\tilde{e}}}_{j}$ **	** ${\bar{\hat{e}}}_{j}$ **	**avg.**	**avg.%**	**s.e.m.**	**min**	**max**
1	134.14	421.94	426.14	−4.20	−1.00%	4.21	−677.54	1083.35
2	137.64	434.06	425.96	8.09	1.86%	4.31	−675.28	1077.25
3	140.10	427.67	425.51	2.16	0.51%	4.26	−692.93	1084.41
4	134.01	421.72	424.59	−2.87	−0.68%	4.31	−658.69	1070.25
5	138.20	432.20	425.59	6.61	1.53%	4.33	−649.86	1067.94
6	134.54	421.22	425.62	−4.40	−1.04%	4.23	−638.90	1067.69
7	138.66	434.07	426.54	7.53	1.74%	4.32	−671.86	1067.94
8	137.66	433.16	426.60	6.56	1.52%	4.31	−676.75	1071.44
9	141.41	430.15	425.71	4.44	1.03%	4.29	−631.85	1067.11
10	140.08	427.10	427.14	−0.04	−0.01%	4.29	−703.10	1072.31

**Table 4:** Results by DoC event from N=10,000 simulations ($N_{c}=9472$) generated by Arm B data.

				Summary Statics of ${\bar{\Delta }}_{j}={\bar{\tilde{e}}}_{j}-{\bar{\hat{e}}}_{j}$				
** j **	** ${\bar{\tilde{\theta }}}_{j}$ **	** ${\bar{\tilde{e}}}_{j}$ **	** ${\bar{\hat{e}}}_{j}$ **	**avg.**	**avg.%**	**s.e.m.**	**min**	**max**
1	170.39	901.20	893.29	7.91	0.88%	8.20	−1245.06	1546.86
2	165.77	903.10	894.93	8.16	0.90%	8.17	−1221.83	1545.27
3	168.02	892.31	891.88	0.43	0.05%	8.16	−1247.44	1527.10
4	168.50	881.61	894.61	−13.00	−1.47%	8.17	−1235.65	1551.53
5	168.56	887.58	893.39	−5.81	−0.65%	8.13	−1248.04	1557.19
6	172.76	889.36	895.64	−6.28	−0.71%	8.11	−1281.15	1545.27
7	167.56	885.83	895.42	−9.59	−1.08%	8.13	−1190.08	1547.59
8	166.83	881.27	895.01	−13.74	−1.56%	8.13	−1271.57	1539.00
9	169.95	886.48	894.43	−7.94	−0.90%	8.18	−1283.04	1547.59
10	167.30	888.51	892.08	−3.57	−0.40%	8.20	−1247.83	1550.47

**Table 5:** Overall results from 10,000 simulations.

	Global Averages of Prediction Errors										
	DoC Imputed							DoC as DoD		DoC as Cen	
**Data**	** $N_{c}$ **	** $\bar{\tilde{\theta }}$ **	** $\bar{\tilde{e}}$ **	** $\bar{\hat{e}}$ **	** $\bar{\Delta }$ **	** $\bar{\Delta }\%$ **	**s.e.m.**	** $\bar{\Delta }$ **	** $\bar{\Delta }\%$ **	** $\bar{\Delta }$ **	** $\bar{\Delta }\%$ **
Arm A	9802	137.64	428.33	425.94	2.39	0.56%	1.338	4.97	1.16%	−20.28	−4.74%
Arm B	9472	168.56	889.72	894.07	−4.34	−0.49%	2.557	−12.65	−1.42%	−63.64	−7.16%

**Table 6:** Effect of CoDMI adjustment for censoring when all COVID-19 endpoints are simulated as censored ($\delta_{j}\equiv 0$). Overall results from 10,000 simulations.

	Global Averages of Prediction Errors											
	DoC Imputed								DoC as DoD		DoC as Cen	
**Data**	**Adjust.**	** $N_{c}$ **	** $\bar{\tilde{\theta }}$ **	** $\bar{\tilde{e}}$ **	** $\bar{\hat{e}}$ **	** $\bar{\Delta }$ **	** $\bar{\Delta }\%$ **	**s.e.m.**	** $\bar{\Delta }$ **	** $\bar{\Delta }\%$ **	** $\bar{\Delta }$ **	** $\bar{\Delta }\%$ **
Arm A	NO	9804	137.55	1004.22	426.09	578.13	57.57%	1.377	579.94	57.80%	554.64	55.28%
	YES	9804	137.55	1004.22	1001.91	2.30	0.23%	1.212	579.94	57.80%	554.64	55.28%
Arm B	NO	9459	168.62	1394.29	894.01	500.28	35.88%	2.119	488.74	35.14%	437.73	31.47%
	YES	9459	168.62	1394.29	1396.58	−2.29	−0.16%	1.899	488.74	35.14%	437.73	31.47%

## Data Availability

Not applicable.

## Appendix A. Derivation of the Extended Greenwood’s Formula

We organize data in a life table with *K* time intervals k=1,2,⋯,K, spanning the interval $\left[0,t_{n+m}^{\prime }\right]$, each with length $\Delta t=t_{n+m}^{\prime }/K$. Let us denote by $h_{k}^{\prime }$ and $\nu_{k}$ the hazard rate and the number of DoD events, respectively, in the interval *k* and by $n_{k}$ the number of subjects at risk at the end of the interval k−1. In this setting the survival function is defined as $S_{l}^{\prime }=\prod_{k=1}^{l}\left(1-h_{k}^{\prime }\right),\;l=1,2,\cdots ,K$, and an estimate ${\hat{S}}_{l}^{\prime }$ for $S_{l}^{\prime }$ is obtained by plugging in an estimate ${\hat{h}}_{k}^{\prime }$ for $h_{k}^{\prime }$, k=1,2,⋯,l. We make the binomial assumption:

(A1) $$n_{k}\;{\hat{h}}_{k}^{\prime }∼\mathrm{Bin}\left(n_{k},h_{k}^{\prime }\right)\;,\;\mathrm{with}\;h_{k}^{\prime }=h_{k}+H_{k}\;,\;k=1,2,\cdots ,K\;,$$

where the parameters $h_{k}$ and $H_{k}$ are the DoD and the DoC hazard rate, respectively. In addition to this usual assumption, we express $H_{k}$ as the random variable:

(A2) $$H_{k}=\frac{N_{k}}{n_{k}},\;\mathrm{with}\;N_{k}=\sum_{j=1}^{m}1_{\left\{\left(k-1\right)\Delta t<T_{\theta_{j}}\leq k\Delta t\right\}}\;,$$

where, as usual, the random variable $T_{\theta_{j}}$ is the conditional lifetime $T_{0}|\left(T_{0}\geq \theta_{j}\right)$ and $\theta_{j}$ is the time of the *j*-th observed DoC event. The probability distribution of $T_{\theta_{j}}$, however, is not necessarily specified for the moment. Let $\mu_{k}=E\left(N_{k}\right)$ and $\sigma_{k}^{2}=\mathrm{Var}\left(N_{k}\right)$.

In order to derive an approximation of the variance of ${\hat{S}}_{l}^{\prime },\;l=1,2,\cdots ,K$, in the same spirit of Greenwood’s formula we consider the variance of the logarithm:

(A3) $$\mathrm{Var}\left(\log{\hat{S}}_{l}^{\prime }\right)=\mathrm{Var}\left[\sum_{k=1}^{l}\log\left(1-{\hat{h}}_{k}^{\prime }\right)\right]=\sum_{k=1}^{l}\mathrm{Var}\left[\log(1-{\hat{h}}_{k}^{\prime })\right]\;.$$

As for the second equality, it should be noted that the ${\hat{h}}_{k}$ values are not independent, since $n_{k}$ depends on the events in the previous periods. However successive conditional independence, given $n_{k}$ (essentially, a martingale argument), is a sufficient condition for the equality to hold. Now we use the so-called delta-method approximation $\mathrm{Var}\left(\log X\right)≃\mathrm{Var}\left(X\right)/{\left[E\left(X\right)\right]}^{2}$, to obtain:

(A4) $$\sum_{k=1}^{l}\mathrm{Var}\left[\log(1-{\hat{h}}_{k}^{\prime })\right]≃\sum_{k=1}^{l}\frac{\mathrm{Var}(1-{\hat{h}}_{k}^{\prime })}{{\left[E\left(1-{\hat{h}}_{k}^{\prime }\right)\right]}^{2}}\;.$$

By the binomial assumption (A1) we have, for all *k*:

$$E\left({\hat{h}}_{k}^{\prime }|H_{k}\right)=h_{k}+H_{k}\;,$$

and:

$$\mathrm{Var}\left({\hat{h}}_{k}^{\prime }|H_{k}\right)=\frac{1}{n_{k}}\;\left(h_{k}+H_{k}\right)\;\left[1-\left(h_{k}+H_{k}\right)\right]\;.$$

Therefore, for the expectation of ${\hat{h}}_{k}^{\prime }$ we obtain:

(A5) $$E\left({\hat{h}}_{k}^{\prime }\right)=h_{k}+\mu_{k}/n_{k}\;,$$

and for the variance of ${\hat{h}}_{k}^{\prime }$ we have:

$$\begin{array}{cc} \mathrm{Var}({\hat{h}}^{\prime }) & =\mathrm{Var}\left[E({\hat{h}}_{k}^{\prime }|H_{k})\right]+E\left[\mathrm{Var}({\hat{h}}_{k}^{\prime }|H_{k})\right]\\ \; & =\mathrm{Var}\left(h_{k}+H_{k}\right)+E\left[\left(h_{k}+H_{k}\right)\;\left[1-\left(h_{k}+H_{k}\right)\right]/n_{k}\right]\;, \end{array}$$

or, with a little algebra:

(A6) $$\mathrm{Var}\left({\hat{h}}_{k}^{\prime }\right)=\frac{1}{n_{k}}\left(h_{k}+\mu_{k}/n_{k}\right)\;\left[1-(h_{k}+\mu_{k}/n_{k})\right]+\frac{n_{k}-1}{n_{k}}\;\frac{\sigma_{k}^{2}}{n_{k}^{2}}\;.$$

By inserting (A5) and (A6) into (A4) we have:

$$\begin{array}{ll} \mathrm{Var}(\log{\hat{S}}_{l}^{\prime }) & ≃\sum_{k=1}^{l}\frac{\mathrm{Var}(1-{\hat{h}}_{k}^{\prime })}{{\left[E\left(1-{\hat{h}}_{k}^{\prime }\right)\right]}^{2}}\\ & =\sum_{k=1}^{l}\left[\frac{h_{k}+\mu_{k}/n_{k}}{1-(h_{k}+\mu_{k}/n_{k})}\;\frac{1}{n_{k}}+\frac{1}{{\left[1-\left(h_{k}+\mu_{k}/n_{k}\right)\right]}^{2}}\frac{n_{k}-1}{n_{k}}\;\frac{\sigma_{k}^{2}}{n_{k}^{2}}\right]\;. \end{array}$$

Plugging in $h_{k}=\nu_{k}/n_{k}$ and posing ${\bar{h}}_{k}=\left(\nu_{k}+\mu_{k}\right)/n_{k}$, we obtain:

$$\mathrm{Var}\left(\log{\hat{S}}_{l}^{\prime }\right)≃\sum_{k=1}^{l}\left[\frac{{\bar{h}}_{k}}{1-{\bar{h}}_{k}}\;\frac{1}{n_{k}}+\frac{1}{{\left(1-{\bar{h}}_{k}\right)}^{2}}\;\frac{n_{k}-1}{n_{k}}\;\frac{\sigma_{k}^{2}}{n_{k}^{2}}\right]\;.$$

Using the inverse approximation $\mathrm{Var}\left(X\right)≃{\left[E\left(X\right)\right]}^{2}\;\mathrm{Var}\left(\log X\right)$, we finally have:

(A7) $$\mathrm{Var}\left({\hat{S}}_{l}^{\prime }\right)≃{\left({\hat{S}}_{l}^{\prime }\right)}^{2}\;\sum_{k=1}^{l}\left[\frac{{\bar{h}}_{k}}{1-{\bar{h}}_{k}}\;\frac{1}{n_{k}}+\frac{1}{{\left(1-{\bar{h}}_{k}\right)}^{2}}\;\frac{n_{k}-1}{n_{k}}\;\frac{\sigma_{k}^{2}}{n_{k}^{2}}\right]\;.$$

Now, in the life table we take Δt small enough to make each time interval contain at most one time point $t_{i}^{\prime }$. In this limit, if $k_{i}$ denotes the interval containing $t_{i}^{\prime }$, we assume that:

$$1_{\left\{\left(k_{i}-1\right)\Delta t<T_{\theta_{j}}\leq k_{i}\Delta t\right\}}=1_{\left\{T_{\theta_{j}}=t_{i}^{\prime }\right\}}\;,$$

consistently with the fact that, in this setting, $T_{\theta_{j}}$ has discrete distribution with probability masses in the points $t_{i}^{\prime }$. These probabilities are the $q_{i,j}^{*}$ provided by CoDMI. Then, by (A2) and (11), we obtain:

$$E\left(N_{k}\right)=\sum_{j=1}^{m}E\left(1_{\left\{T_{\theta_{j}}=t_{i}^{\prime }\right\}}\right)=\sum_{j=1}^{m}P\left(T_{\theta_{j}}=t_{i}^{\prime }\right)=\sum_{j=1}^{m}\;q_{i,j}^{*}=Q_{i}\;.$$

For the variance, assuming the independence of the $T_{\theta_{j}}$ we have:

$$\mathrm{Var}\left(N_{k}\right)=\sum_{j=1}^{m}\mathrm{Var}\left(1_{\left\{T_{\theta_{j}}\right)=t_{i}^{\prime }\}}\right)=\sum_{j=1}^{m}q_{i,j}^{*}\;\left(1-q_{i,j}^{*}\right)=\sum_{j=1}^{m}\;q_{i,j}^{*}-\sum_{j=1}^{m}\bar{(}q_{i,j}^{*}{)}^{2}=Q_{i}---Q_{i}^{\left(2\right)}\;.$$

Thus, we estimate $\mu_{k}$ by $Q_{i}$ and $\sigma_{k}^{2}$ with $Q_{i}---Q_{i}^{\left(2\right)}$. Putting it all together, for the survival function $S_{l}^{\prime }$ we arrive at the product-limit estimator:

(A8) $${\hat{S}}^{\prime }\left(t\right)=\prod_{i:t_{i}^{\prime }\leq t}\left(1-{\bar{h}}_{i}\right)\;,\;t\geq 0\;,$$

where:

$${\bar{h}}_{i}=\frac{d_{i}^{\prime }\;\nu_{i}}{R_{i}},\;\mathrm{with}\;\nu_{i}=\left(1-\delta_{i}^{\prime }\right)+Q_{i},\;i=1,2,\cdots ,n+m\;,$$

and $R_{i}$ is computed recursively as:

$${\bar{R}}_{1}=n+m,\;{\bar{R}}_{i}={\bar{R}}_{i-1}-\left[1+\left(\nu_{i}-1\right)\;d_{i}^{\prime }\right],\;i=2,3,\cdots ,n+m\;.$$

Correspondingly, (A7) reduces to:

(A9) $$\mathrm{Var}\left({\hat{S}}^{\prime }\left(t\right)\right)≃{\left({\hat{S}}^{\prime }\left(t\right)\right)}^{2}\;\sum_{i:t_{i}^{\prime }\leq t}\left[\frac{{\bar{h}}_{i}}{1-{\bar{h}}_{i}}\;\frac{1}{{\bar{R}}_{i}}+\frac{1}{{\left(1-{\bar{h}}_{i}\right)}^{2}}\;\frac{{\bar{R}}_{i}-1}{{\bar{R}}_{i}}\;\frac{Q_{i}-Q_{i}^{\left(2\right)}}{{\bar{R}}_{i}^{2}}\right]\;.$$

In summary, the addition of the random component in the binomial assumption (A1) has the effect of distributing each of the *m* COVID-19 deaths, which has been imputed by CoDMI on the time points ${\hat{\tau }}_{j}^{*}$, on all the uncensored time points according to its truncated distribution $q_{i,j}^{*}$. Summing over *j* we obtain the total probabilities $Q_{i}$, for which the property holds $\sum_{i}Q_{i}=m$. In the variance expression (A9) the estimation error of the ${\hat{\tau }}_{j}^{*}$ is taken into account by the additional term containing the variance estimates $Q_{i}-Q_{i}^{\left(2\right)}$.

It should be noted, however, that the survival function estimate ${\hat{S}}^{\prime }\left(t\right)$ given by (A8) is slightly different by the estimate $\hat{S}\left(t\right)$ given by (1). Since the COVID-19 deaths are spread out on all the time points, one usually has ${\hat{S}}^{\prime }\left(t\right)\geq \hat{S}\left(t\right)$ for small *t* and ${\hat{S}}^{\prime }\left(t\right)\leq \hat{S}\left(t\right)$ for large *t*. One can accept the approximation:

(A10) $$\mathrm{Var}\left({\hat{S}}^{\prime }\left(t\right)\right)≃{\left(\hat{S}\left(t\right)\right)}^{2}\;\sum_{i:t_{i}^{\prime }\leq t}\left[\frac{{\bar{h}}_{i}}{1-{\bar{h}}_{i}}\;\frac{1}{{\bar{R}}_{i}}+\frac{1}{{\left(1-{\bar{h}}_{i}\right)}^{2}}\;\frac{{\bar{R}}_{i}-1}{{\bar{R}}_{i}}\;\frac{Q_{i}-Q_{i}^{\left(2\right)}}{{\bar{R}}_{i}^{2}}\right]\;,$$

which gives Formula (12). The more conservative approximation:

(A11) $$\mathrm{Var}\left({\hat{S}}^{\prime }\left(t\right)\right)≃{\left(\max\{\hat{S}\left(t\right),{\hat{S}}^{\prime }\left(t\right)\}\right)}^{2}\;\sum_{i:t_{i}^{\prime }\leq t}\left[\frac{{\bar{h}}_{i}}{1-{\bar{h}}_{i}}\;\frac{1}{{\bar{R}}_{i}}+\frac{1}{{\left(1-{\bar{h}}_{i}\right)}^{2}}\;\frac{{\bar{R}}_{i}-1}{{\bar{R}}_{i}}\;\frac{Q_{i}-Q_{i}^{\left(2\right)}}{{\bar{R}}_{i}^{2}}\right]\;,$$

could be also considered.
